# Lipid Peroxidation and Paraoxonase-1 Activity in Celiac Disease

**DOI:** 10.1155/2012/587479

**Published:** 2012-03-25

**Authors:** Gianna Ferretti, Tiziana Bacchetti, Letizia Saturni, Nicola Manzella, Cinzia Candelaresi, Antonio Benedetti, Antonio Di Sario

**Affiliations:** ^1^Dipartimento di Scienze Cliniche Sperimentali e Odontostomatologiche, Università Politecnica delle Marche, 60131 Ancona, Italy; ^2^Dipartimento di Scienze della Vita e dell'Ambiente, Università Politecnica delle Marche, 60131 Ancona, Italy; ^3^Clinica di Gastroenterologia, Università Politecnica delle Marche, 60131 Ancona, Italy

## Abstract

Paraoxonase-1 (PON1) plays an antioxidant and anti-inflammatory role. Aim of the study was to investigate the alteration of paraoxonase-1 activity in celiac disease (CD), an intestinal disorder characterized by toxic injury exerted by gluten peptides. Activities of PON1, levels of biochemical markers of lipid peroxidation and total antioxidant capacity were evaluated in serum obtained from 27 celiac patients (11 at diagnosis, 16 treated with gluten free diet) and 25 healthy subjects. Moreover, the serum susceptibility of Cu^2+^-induced lipid peroxidation was investigated in controls and patients. The results showed a lower PON1 activity in serum of both groups of celiac patients with respect to control subjects. PON1 activity in CD was related with markers of disease severity and was negatively correlated with the levels of lipid hydroperoxide and with the susceptibility of serum to lipid peroxidation induced in vitro by metal ions. The alteration of PON1 activity and markers of lipid peroxidation realized at lower extent in patients who were on a gluten-free diet.

## 1. Introduction

Environmental, genetic, and immunologic factors are involved in the pathogenesis of celiac disease (CD) [1], a chronic inflammatory disease characterised by flattened villi on the small bowel mucosa. CD is induced by the ingestion of cereals containing proline-rich and glutamine-rich proteins (such as wheat, rye, and barley) in genetically susceptible individuals [2, 3]. From a clinical point of view, CD is characterised by a clinical heterogeneity that ranges from asymptomatic to severely symptomatic and by increased morbidity and mortality [4]. At present, the only proven treatment for CD is strict and life-long adherence to a gluten-free diet (GFD) [1–4].

An increase of markers of oxidative damage of lipids (thiobarbituric acid-reactive substances and lipid hydroperoxides) and proteins (carbonyl groups) and a decrease of antioxidant enzymes have been demonstrated in plasma, in circulating cells and in intestinal cells of patients with CD with respect to controls [5–8]. Higher levels of nitric oxide (NO) and biochemical markers of oxidative damage of DNA has been demonstrated in leukocytes and in urine samples of celiac children [9, 10], suggesting that oxidative stress could be involved in the pathogenesis of CD.

Paraoxonase-1 (PON1) is an enzyme associated with the high-density lipoproteins (HDL) that plays both an antioxidant and an anti-inflammatory role [11–13]. A decrease in PON1 has been reported in several human diseases associated with inflammation and alteration of lipoprotein metabolism [14–17]. Interest in the study of PON1 in celiac disease is supported by recent studies that have suggested a possible role of PON1 in inflammatory intestinal diseases [18–20]. Furthermore it has been hypothesised that in the gastrointestinal tract, PON1 could behave as a local detoxifier, antioxidant, immunomodulator, and/or quorum-quenching factor [18].

To further investigate the relationship between oxidative damage and CD, we studied the levels of lipid peroxides, total antioxidant capacity, the susceptibility to copper-induced lipid peroxidation, and the activity of the enzyme paraoxonase-1 (PON1) in serum of three groups of subjects: untreated patients with a new diagnosis of celiac disease (CD patients), CD patients on a gluten-free diet (GFD patients), and healthy subjects.

## 2. Materials and Methods

### 2.1. Subjects Included in the Study

The study included 27 consecutive patients who were referred to the Celiac Disease Clinic of the Department of Gastroenterology of the Polytechnic University of Marche, and the study protocol was in accordance with the ethical standards formulated in Helsinki Declaration as revised in 2000. Eleven patients (3 males and 8 females, mean age 31.2 ± 11.7 years) had a new diagnosis of CD (CD patients), whereas 16 (GFD patients; 7 males and 9 females, mean age 35.8 ± 12.1 years) were celiac patients on a gluten-free diet (mean duration: 19.2 ± 5.7 months). Twenty-five subjects (12 males and 13 females, mean age 39.6 ± 8.1 years) who underwent esophagogastroduodenoscopy for the presence of dyspeptic syndrome and who did not result to be affected by CD were used as controls. Subjects with diabetes and clinical evidence of cardiovascular diseases or receiving antacids, lipid-lowering drugs, or antioxidant supplements were excluded from the study to avoid possible interferences on PON1 activity and plasma lipids.

All subjects included in the study underwent upper gastrointestinal endoscopy and at least 4–6 well-oriented duodenal specimens were taken for the histological evaluation.

The degree of intestinal mucosal damage was then classified according to the Marsh classification [21]. Thereafter, each stage was given a score from 0 (normal mucosa) to 5 (total villous atrophy) for statistical analysis.

Serum levels of antitransglutaminase(t-TG) antibodies were assessed by the laboratory of our institution by using a commercial FluoroEnzymeImmuno Assay kit (Phadia, Sweden) and the results were expressed as unit (U)/mL of serum. A value higher than 10 U/mL was considered positive [22].

In the same day of endoscopy, blood drawn by venous puncture by fasted subjects was collected into serum-separator tubes. After clotting, blood was centrifuged for 15 min at 3500 rpm to separate serum that was stored at −80°C until examination.

### 2.2. Serum PON1 Activities

PON1 activity has been evaluated using three different substrates [23]: paraoxon for paraoxonase activity, phenylacetate for arylesterase activity, and dihydrocoumarin for lactonase activity. All assays were performed in a 96-well plate, in a total reaction volume of 200 *μ*L.

#### 2.2.1. Paraoxonase Activity

Serum (10 *μ*L, nondiluted samples) were analyzed. The basal assay mixture included 5 mM Tris-HCl, pH 7.4 containing 0.15 M NaCl, 4 mM MgCl_2_, 2 mM CaCl_2_, and 1.0 mmol/L paraoxon. Paraoxon hydrolysis was spectrophotometrically monitored for 8 min (every 15 s) at 412 nm. Nonenzymatic hydrolysis of paraoxon was subtracted from the total rate of hydrolysis. One unit of PON1 paraoxonase activity was equivalent to 1 nmol of paraoxon hydrolyzed/min/mL [23].

#### 2.2.2. Arylesterase Activity

The serum samples were diluted 1 : 10 with 1 mmol/L CaCl_2_ in 50 mmol/L Tris HCl, pH 8.0 and then, 5 *μ*L was taken for a total reaction volume of 200 *μ*L. After addition of the substrate phenyl acetate (1.0 mmol/L), the hydrolysis was monitored at 270 nm for 3 min (every 15 s). One unit of arylesterase activity was equivalent to 1 *μ*mol of phenyl acetate hydrolyzed/min/mL [23].

#### 2.2.3. Lactonase Activity toward Dihydrocoumarin (DHC)

The serum samples were diluted 1 : 10 with 1 mmol/L CaCl_2_ in 50 mmol/L Tris HCl, pH 8.0 and 3 *μ*L was then taken for the assay. After addition of the substrate DHC (1.0 mmol/L), the hydrolysis was monitored at 270 nm for 10 min (every 15 s). Nonenzymatic hydrolysis of DHC was subtracted from the total rate of hydrolysis. One unit of lactonase activity was equivalent to 1 *μ*mol of DHC hydrolyzed/min/mL [23].

### 2.3. Serum Oxidative Stress Parameters

#### 2.3.1. Serum Basal Oxidative Stress Status

Basal serum oxidative status was determined by the evaluation of the levels of lipid hydroperoxides using FOX2 assay [15]. Briefly, an aliquot (200 *μ*L) of serum was mixed with 1800 *μ*L of FOX2-reagent (250 *μ*mol/L ammonium ferrous sulphate, 100 *μ*mol/L xylenol-orange, 25 mmol/L H_2_SO_4_ and 4 mmol/L BHT in 90% methanol (v/v) in 100 mL). After incubation at room temperature for 30 minutes, samples were centrifuged at 4000 rpm for 10 minutes. The supernatant was carefully decanted into a cuvette and the absorbance was determined at 560 nm. The levels of lipid hydroperoxides were quantified using a stock solution of t-butyl hydroperoxide. The results were expressed as *μ*mol of lipid hydroperoxides for L of serum.

#### 2.3.2. Copper-Induced Serum Lipid Peroxidation

Serum samples were diluted 4x with phosphate-buffered saline (PBS-citrate) and were incubated with 50 *μ*mol/L CuSO_4_. Serum lipid peroxidation was monitored by following the kinetic of conjugated dienes formation at 234 nm [24]. The increase of absorbance was recorded for 6 hours. A KC4 software was used to calculate the lag times of kinetic of conjugated dienes formation. The lag time (t-lag, min), defined as the intercept of the straight lines derived from the lag phase and the propagation phase, is an index for resistance of serum to lipid peroxidation [24]. As previously demonstrated longer lag time indicates a lower susceptibility of serum to lipid peroxidation [24].

### 2.4. Serum Total Antioxidant Capacity

Serum total antioxidant capacity (TAC) was assessed using the oxygen radical absorbance capacity (ORAC) assay described by Ou et al. [25]. The oxygen radical absorbance capacity of serum employs the oxidative loss of the intrinsic fluorescence of fluorescein induced by the free radical initiator 2,2′-azobis(2-amidinopropane)hydrochloride (AAPH). Trolox was used as a reference antioxidant for calculating the ORAC values. Results were expressed as *μ*mol Trolox equivalents/L (*μ*mol TE/L).

### 2.5. Statistical Analysis

Data were reported as mean ± standard deviation (SD). PON1 activities were presented as medians. For the comparison of normally distributed variables between groups, Student's *t*-test was used. Paraoxonase-1 activity showed a non-Gaussian distribution; therefore we used a nonparametric test (Wilcoxon rank sum test). Spearman's correlation coefficient was used to test the strength of any association between different variables. All tests were 2-tailed and a *P* < 0.05 level of significance was used to assess statistical significance (Microcal Origin 5.0, OriginLab, Northampton, MA).

## 3. Results

### 3.1. Clinical Characteristics of the Study Population

As shown in Table 1, serum levels of anti-t-TG antibodies were within the normal range in healthy subjects (0.4 ± 0.2 U/mL). As expected, significant higher levels were observed in untreated celiac patients (108.2 ± 35.9 U/mL). In GFD patients the levels of anti-t-TG antibodies were significantly higher compared with controls but significantly lower with respect to untreated CD patients (Table 1). The degree of intestinal damage was in the order: controls < GFD patients < CD patients (Table 1). As summarized in Table 1, the differences were statistically significant.

### 3.2. Plasma Lipids in Controls and Celiac Patients

The mean levels of serum total cholesterol (TC), HDL-cholesterol and LDL-cholesterol, and triglycerides (TG) were lower in CD patients with respect to controls, even if the differences were not statistically significant (Table 1). The levels of plasma lipids in GFD patients were higher with respect to CD patients, although the differences were not statistically significant (Table 1).

### 3.3. Serum Paraoxonase-1 Activities in Controls and Celiac Patients

As summarized in Table 2, the median values of PON1 paraoxonase activity were in the order controls > GFD patients > CD patients. The differences between both groups of CD patients compared with controls were statistically significant (Table 2). The median values of lactonase and arylesterase activities of PON1 in serum of CD and GFD patients were significantly lower with respect to controls (Table 2). PON1 paraoxonase activity was significantly correlated with arylesterase (*r* = 0.871, *n* = 52, *P* < 0.0001) and lactonase (*r* = 0.875, *n* = 52, *P* < 0.0001) activities.

### 3.4. Serum Lipid Peroxidation Markers and Total Antioxidant Capacity in Controls and Celiac Patients

The levels of lipid hydroperoxides in control subjects were 2.02 ± 0.76 *μ*mol/L. Higher levels of lipid hydroperoxides were observed in serum of CD and GFD patients with respect to controls. In serum of GFD patients, the values were significantly lower compared with untreated CD patients (Table 2).

To investigate serum susceptibility to lipid peroxidation in celiac patients, we evaluated the formation the conjugated dienes in serum oxidized “*in vitro*” with copper ions. The mean value of the lag time of the kinetic of lipid peroxidation in serum of controls was 66.4 ± 16.2 minutes (Table 2). The lag time was significantly lower in serum of CD and GFD patients. In GFD patients the lag time was significantly higher with respect to CD patients (Table 2).

As summarized in Table 2, in GFD patients serum TAC was lower than controls and significantly higher than that of CD patients.

### 3.5. Relationship between PON1 Paraoxonase Activity and Clinical Parameters in Celiac Patients

To study whether the activity of paraoxonase-1 and markers of oxidative stress were related to severity of CD, we compared the mean values of serum levels of anti-t-TG and the degree of mucosal damage (following Marsh index) in two subgroups of celiac patients. The first group included 14 CD patients having PON1 paraoxonase activity below the median value of PON1 observed in all celiac patients (124.6 U/mL) (10 untreated CD and 4 GFD patients). The second group included 13 patients with PON1 activity above the median value (1 untreated CD patient and 12 GFD patients).

**Figure 1:**
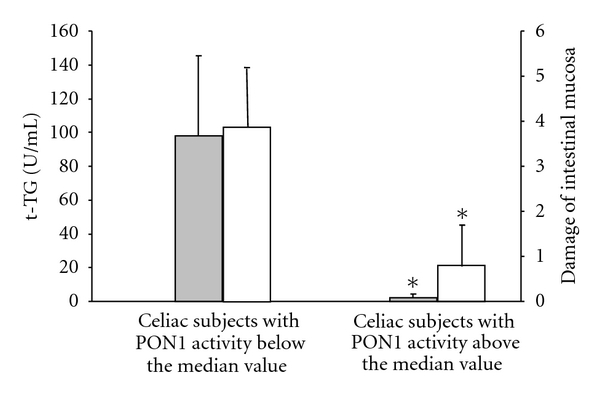
Mean levels of antitransglutaminase(t-TG) antibodies levels (grey square) and degree of damage of intestinal mucosa classified according to Marsh parameters (white square) in celiac patients with PON1 activity above or below the median value (124.6 U/mL). *P < 0.001 versus celiac patients with PON1 activity below median value.

As shown in Figure 1, the mean levels of serum anti-t-TG and the degree of mucosal damage were significantly higher in celiac patients with serum PON1 activity below the median value with respect to patients with PON1 activity above the median value. The differences were statistically significant.

Similar results were observed also for PON1 arylesterase and lactonase activities (data not shown).

### 3.6. Correlations between PON1 Paraoxonase Activity and the Other Markers of Oxidative Stress

**Figure 2:**
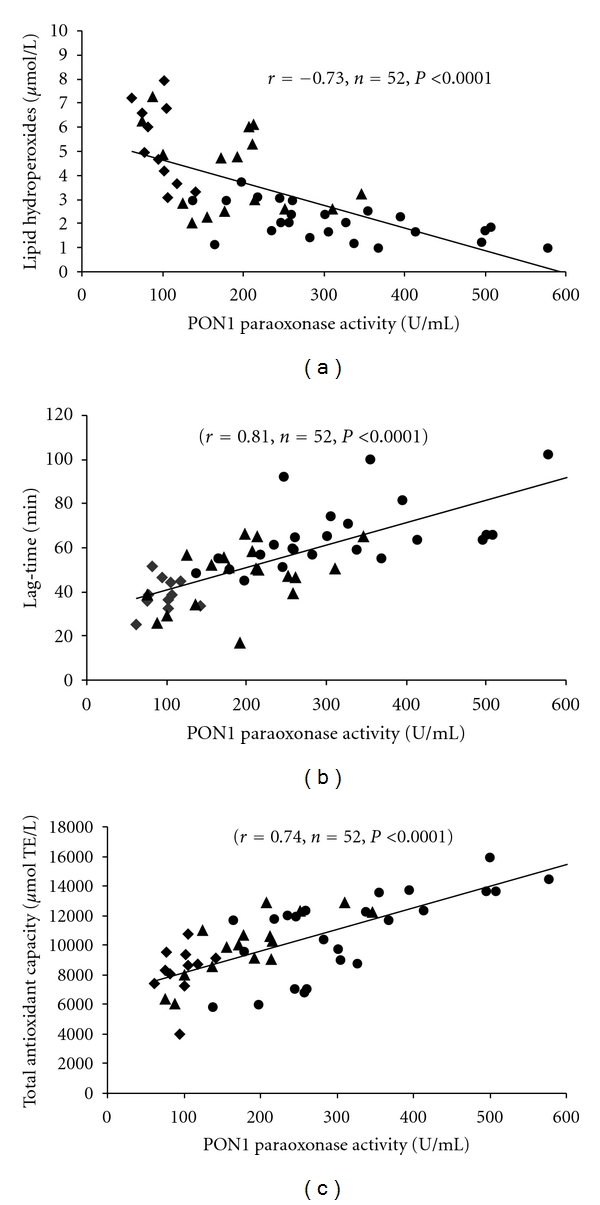
Correlations between PON1 paraoxonase activity versus the concentrations of serum lipid hydroperoxides (a), versus duration of the lag-time of Cu^2+^-oxidized serum (b), versus serum total antioxidant capacity (c) of controls (•) and untreated (♦) or GFD-treated (▲) celiac patients.

The individual values of PON1-paraoxonase activity and the levels of serum hydroperoxides were negatively correlated (*r* = −0.735, *n* = 52, *P* < 0.0001), in agreement with our previous studies [15] (Figure 2(a)). In the whole group of subjects included in the study, the enzyme activity was positively correlated with the lag time of kinetic of lipid peroxidation (*r* = 0.811, *n* = 52, *P* < 0.0001) (Figure 2(b)) and with the values of total antioxidant capacity (*r* = 0.74, *n* = 52, *P* < 0.0001) (Figure 2(c)). Levels of lipid hydroperoxides were negatively correlated with total antioxidant capacity (*r* = −0.52, *n* = 52, *P* < 0.0001) and lag-time values (*r* = −0.59,  *n* = 52,  *P* < 0.0001).

No significant correlation was observed between PON1 activity and HDL cholesterol levels (data not shown).

## 4. Discussion

The clinical interest in human PON1 has been mainly focused on atherosclerosis and cardiovascular disease [14, 26], since PON1 exerts antioxidant and anti-inflammatory roles [14–16]. However, more recently a possible role of PON1 in the human small intestine in normal and pathological conditions has been suggested [18–20].

Although PON1 paraoxonase activity has been manly investigated in normal and pathological conditions using paraoxon as substrate [15–17], we evaluated also PON1 arylesterase and lactonase activities because previous studies have suggested that serum arylesterase activity could reflect serum enzyme concentration [27] and lactonase activity could represent a more physiological activity of the enzyme [28].

Our results demonstrated, for the first time, a significant decrease of PON1 activities (paraoxonase, arylesterase, and lactonase) in serum of celiac patients.

The modifications of PON1 activities in celiac patients were related with the severity of the disease and were associated with an increase of lipid hydroperoxide levels and with a higher susceptibility of serum to lipid peroxidation induced “in vitro.” Moreover, a lower serum total antioxidant capacity (TAC) has been shown in celiac patients. The values of PON1 activity, markers of lipid peroxidation, and total antioxidant capacity in GFD patients were significantly different compared with healthy subjects and untreated CD patients.

Our results confirm that CD is associated with oxidative damage as suggested in previous studies mainly carried out in CD children [6, 8–10].

**Figure 3:**
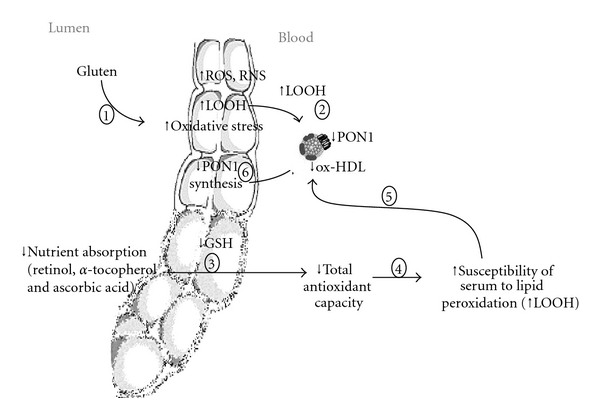
Possible molecular mechanisms involved in increased lipid peroxidation and decreased PON1 activity in celiac disease. Gluten ingestion induces an increased oxidative stress, due to overproduction of free radicals (ROS and RNS) (1). The higher levels of ROS can trigger lipid peroxidation (LOOH) in intestinal mucosa and blood (2). Oxidative stress could be favored by lower serum antioxidant capacity resulting from lower absorption of dietary antioxidants due to mucosal damage (3). All the aforementioned changes could explain the higher susceptibility of serum to lipid peroxidation (4) and the compositional alterations of HDL with consequent decrease of PON1 activities (5). In addition to the inhibition exerted by lipid peroxidation products, the lower PON1 activity could be mediated by inhibition of PON1 synthesis from gastrointestinal cells (6).

Some hypotheses can be made to explain the molecular mechanisms involved in the lower PON1 activity and the higher oxidative stress in CD patients (Figure 3).

Previous studies have demonstrated that, in CD patients, gluten ingestion induces an increased oxidative stress due to overproduction of free radicals (ROS and RNS) [29–31]. The higher levels of ROS and the decrease of dietary antioxidant can trigger lipid peroxidation (LOOH) in intestinal mucosa and blood lipoproteins.

PON1 is a lipid-dependent enzyme associated with high-density lipoprotein (HDL) [32]. Previous studies demonstrated that a decrease of serum PON1 activity could be related to a lower hepatic synthesis and secretion or to inhibition exerted by lipid hydroperoxides [27].

No significant correlation has been established between PON1 activity and levels of HDL cholesterol, suggesting that the lower enzyme activity in CD patients is not associated with alterations of HDL levels.

Although PON1 is mainly synthesised by the liver, it has been demonstrated that it is expressed also in human gastrointestinal tract and Caco-2 cell lines, suggesting that the intestine, as well as the liver, could represent a source for circulatory PON1 [33]. Since a decreased expression of PON1 mRNA has been reported by Rothem L et al. in intestinal biopsies of celiac patients [18], we could speculate that the lower PON1 activity could be related to changes of the enzyme synthesis from the gastrointestinal tract. However, contrasting results have been reported on the synthesis of PON1 in intestinal cells [18, 34–36].

The lower activity of PON1 in CD patients could be also mediated by inhibition exerted by lipid peroxidation products, since previous studies have shown that PON1 paraoxonase, arylesterase, and lactonase activities are inhibited by lipid peroxidation products [37, 38]. This hypothesis is supported by the significant negative correlation found between PON1 paraoxonase activity and levels of lipid hydroperoxides in serum of controls and celiac patients.

The higher susceptibility of serum of celiac patients to Cu^2+^-triggered lipid peroxidation, demonstrated in our experimental conditions, may be related to the lower PON1 activity and/or to the lower serum total antioxidant capacity, as shown by the significant correlations observed between the lag time of kinetic of lipid peroxidation and PON1 paraoxonase values and TAC values.

The lower serum total antioxidant capacity in CD patients could be likely the result of a malabsorption of dietary antioxidants due to the mucosal damage. In agreement with this hypothesis other authors have reported lower levels of antioxidants (retinol, *α*-tocopherol, and ascorbic acid) in plasma of CD patients [5, 9]. The mean level of serum total antioxidant capacity in GFD patients was significantly higher compared with untreated CD patients, although it did not reach the values observed in controls; these results suggest that gluten-free diet is able to only partially improve the functionality of intestinal mucosa.

Whatever are the causes of the decrease in the activity of the antioxidant and anti-inflammatory enzyme PON1, our results might have a physiopathological relevance. In fact, the enzyme PON1 exerts several physiological roles. In the gastrointestinal tract PONs proteins and could behave as detoxifier against food antigens, antioxidant against exogenous and endogenous lipid peroxides, immunomodulators and/or inhibitors of bacterial quorum-quenching factor [18, 35]. Moreover, PON1 exerts a protective effect against lipid peroxidation of lipoproteins and biological membranes [11–13]. Oxidative stress is one of the main causes of damage to cell membranes and lipoproteins. End products of lipid peroxidation (aldehydes, lipid peroxides) react with biological macromolecules such as DNA and proteins and can cause changes in intestinal cell membrane structure and properties leading to loss of its integrity [39]. Moreover, lipid peroxidation products exert biological effects, modulate signal transduction and it has been proposed that they may have a role in inflammatory processes [39].

In conclusion, our results confirm that celiac disease is associated with oxidative damage and with significant decrease of PON1 activities. Although a decrease of paraoxonase-1 activity and an increase of lipid peroxidation products are not specific of celiac disease [14–17], they could exert a proinflammatory and cytotoxic effect and could therefore contribute to gastrointestinal cell injury in CD.

## Figures and Tables

**Table 1:** Clinical characteristics and levels of serum lipids in healthy subjects, celiac patients at diagnosis (CD patients) and celiac patients treated with a gluten-free diet (GFD patients). Data are given as means ± S.D.

	Healthy subjects (*n* = 25)	Celiac patients (*n* = 27)	
		CD patients(*n* = 11)	GFD patients (*n* = 16)
Serum t-TG^a^ (U/mL)	0.4 ± 0.2	108.2 ± 35.9*	2.3 ± 1.9^∗#^
Damage of intestinal mucosa^b^	0	4.4 ± 0.6*	1.4 ± 0.7^∗#^
Serum triglycerides (mg/dL)	87.140.5±	61.3 ± 23.5	71.2 ± 48.2
Serum cholesterol (mg/dL)	186.6 ± 43.4	154.1 ± 27.6	177.1 ± 42.6
Serum HDL-cholesterol (mg/dL)	56.3 ± 13.4	51.1 ± 7.9	54.6 ± 8.7
Serum LDL-cholesterol (mg/dL)	113.2 ± 23.7	90.6 ± 16.1	108.8 ± 26.2

^a^t-TG: antitransglutaminaseantibodies. ^b^Damage of intestinal mucosa was classified according to Marsh parameters and each stage was given a score from 0 (normal mucosa) to 5 (total villous atrophy).

^∗^ *P* < 0.001 versus healthy subjects; ^#^ *P* < 0.05 versus CD patients.

**Table 2:** PON1 activities, markers of lipid oxidative stress (levels of lipid hydroperoxides and lag time of conjugated dienes) and total antioxidant capacity (TAC) in serum of healthy subjects, celiac patients at diagnosis (CD patients), and celiac patients treated with a gluten-free diet (GFD patients). Data are given as median (range) for paraoxonase activities and means ± S.D. for other biochemical parameters.

	Healthy subjects (*n* = 25)	Celiac patients (*n* = 27)	
		CD patients (*n* = 11)	GFD patients (*n* = 16)
PON1 Activities			
Paraoxonase (U/mL)	306 (165.6–608.1)	101.5 (61.2–141.6)*	184.9 (75.3–347.8)^∗#^
Lactonase (U/mL)	68 (32–184)	34 (19.7–68.5)*	54.7 (16.5–101.6)^∗#^
Arylesterase (U/mL)	228 (90–612)	73.2 (41.2–109.7)*	105.5 (68.2–148.7)^∗#^
Lipid hydroperoxides (*μ*mol/L)	2.02 ± 0.76	5.31 ± 1.62*	4.14 ± 1.50^∗#^
Lag time (min)	66.4 ± 16.2	39.4 ± 7.2*	46.7 ± 12.8^∗#^
TAC (*μ*M TE)	12048 ± 2678	8796 ± 976*	9977 ± 1021^∗#^

^∗^ *P* < 0.001 versus controls; ^#^ *P* < 0.05 versus CD patients.
